# Gating-spring stiffness increases outer-hair-cell bundle stiffness, damping, and receptor current

**DOI:** 10.1038/s41598-024-81355-5

**Published:** 2024-12-02

**Authors:** Zenghao Zhu, Wisam Reid, Dáibhid Ó Maoiléidigh

**Affiliations:** 1 Department of Otolaryngology-Head and Neck Surgery, Stanford University, Stanford, 94304 CA USA; 2 Harvard Medical School, Harvard University, Boston, 02114 MA USA

**Keywords:** Mechanotransduction, Cell mechanics, Hearing, Balance, Hair bundle, Hair cell, Biological physics, Cochlea, Hair cell, Transduction

## Abstract

In our ears, outer-hair-cell bundles (OHBs) convert sound-induced forces into receptor currents that drive cochlear amplification, the process responsible for the micropascal-scale threshold and million-fold dynamic range of hearing. OHBs rely on gating springs to open mechanoelectrical-transduction (MET) ion channels, through which the receptor current flows. OHBs have larger gating-spring stiffnesses than other types of hair bundles, but we have a poor understanding of how gating-spring stiffness contributes to OHB mechanics and receptor-current regulation. Using experimentally-constrained mathematical models of the OHB, we show that the increased gating-spring stiffness in an OHB increases its stiffness and damping. The OHB’s 3D morphology reduces the contribution of gating-spring stiffness to OHB stiffness, reduces the contribution of MET-channel gating to OHB stiffness and damping, but causes additional OHB damping that rises with gating-spring stiffness. Gating-spring stiffness increases the OHB’s receptor current but decreases its displacement-current dynamic range. Strikingly, the OHB’s 3D morphology causes its force-current dynamic range to decrease with gating-spring stiffness. Our results suggest a trade-off between threshold and dynamic range regulated by OHB gating-spring stiffness.

## Introduction

Our senses of hearing and balance rely on sensory hair bundles, which convert forces caused by sound, head position, or head acceleration into electrical currents — a process called mechanoelectrical transduction (MET)^1^. Hair-bundle mechanics differs in distinct species, organs, locations within an organ, and cell types. How specializations in hair-bundle mechanics affect sensory performance in these different contexts is a fundamental unresolved question.

**Fig. 1:**
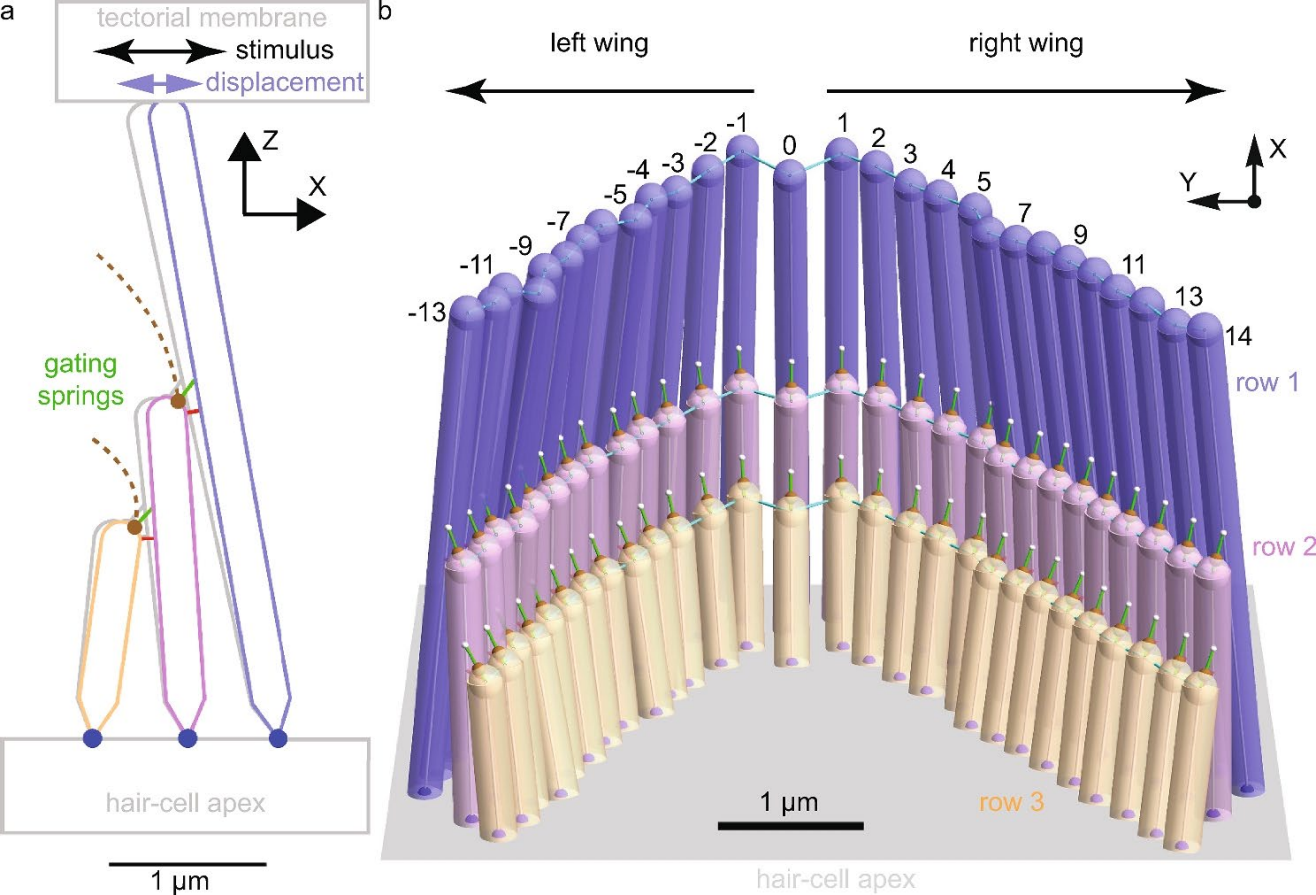
OHBs transduce sound-induced forces into receptor currents. (**a**) An OHB column is shown, comprising three stereocilia of differing heights (row 1 in blue, row 2 in purple, and row 3 in orange) emanating from the hair-cell apex (gray) and linked by gating springs (green) and connectors (red). When the overlying tectorial membrane (gray) applies a stimulus force (black) to the row-1 stereocilium, the stereocilia deflect (gray/color), pivoting at their insertion points in the hair-cell apex (blue dots), and the gating springs extend. Consequently, MET-channels (brown dots) at the lower ends of the gating springs open and close modulating the currents (brown dashed lines) entering the hair cell. The receptor current is the sum of these currents. (**b**) A complete OHB with mutant gating springs (one tenth the stiffness of wild-type) is shown, comprising twenty-eight columns of stereocilia. Columns are numbered relative to the central column labeled 0. Left (negative numbers) and right (positive numbers) wings are indicated by arrows. Stereocilia of similar height form rows and are linked by connectors (cyan).

Hair bundles comprise stereocilia, filamentous rods protruding from the surface of their sensory hair cell (Fig. 1a). Stereocilia of differing height are linked by tip links, a dimer of protocadherin 15 attached to a dimer of cadherin 23^2^. Tip-links and other molecules in series with them (e.g., LHFPL5) form gating springs, which apply forces on MET ion channels in the shorter stereocilia^3,4^. Mutations in gating springs cause bundle dysfunction and hearing loss, but how gating-spring changes cause bundle dysfunction is poorly understood^3,5^.

Stimuli toward the taller stereocilia cause all stereocilia to pivot around their insertion point in the hair-cell apex, extending the gating springs and opening the MET channels. The sum of the currents entering the hair cell through the MET channels is called the receptor current.

In experiments, the stimulus force applied to the bundle can be estimated and the tip displacement of the tallest stereocilia in the direction of the stimulus can be measured^6–9^. The magnitude of the stimulus force relative to the displacement is called the bundle’s stiffness. When gating springs are chemically cut, the bundle’s stiffness decreases, implying that gating springs contribute to bundle stiffness^9^. Gating-spring stiffness is calculated from these data using mathematical models that explain how gating-spring stiffness relates to bundle stiffness^7–10^.

Gating-spring stiffness is correlated with sensory role. Hair bundles in the frog sacculus (used for balance and low-frequency sound and vibrations) detect low-frequency (< 200 Hz) stimuli and have small gating-spring stiffnesses (0.6 mN/m)^8^. In the mammalian hearing organ, the cochlea, outer-hair-cell bundles (OHBs) and inner-hair-cell bundles in rats can detect higher-frequency stimuli (up to 71 kHz) and can have larger gating-spring stiffnesses (up to 8 mN/m)^9–11^. Along the cochlea, the gating-spring stiffness of OHBs and inner-hair-cell bundles increases with their characteristic frequency (the stimulus frequency evoking the largest response to low-level sound at each cochlear location *in vivo*)^9^. However, at the same characteristic frequency, OHBs have larger gating-spring stiffnesses than inner-hair-cell bundles^9^. OHB receptor currents drive the processes in outer hair cells that amplify sound-induced mechanical vibrations in the cochlea, whereas the receptor currents of inner-hair-cell bundles drive the processes that cause auditory neurons to fire^12,13^. Larger gating-spring stiffnesses in OHB may be associated with their role in cochlear amplification, but the effects of increased gating-spring stiffness in OHBs are unknown.

Mechanical amplification by outer hair cells is responsible for the low threshold and wide dynamic range of mammalian hearing^12,13^. OHB mechanics contributes to cochlear mechanics, the OHB receptor current drives amplification, and the dynamic range of the OHB receptor current underlies the dynamic range of mammalian hearing^14–18^. Controlling the mechanics and receptor-current of OHBs may provide a means of regulating cochlear amplification.

OHBs comprise columns of stereocilia (stereocilia of differing height linked by gating springs) and three rows of stereocilia (stereocilia of similar height) (Fig. 1). The columns of stereocilia are far from parallel and the tallest row (row 1) is V shaped^10^. Neighboring stereocila are also coupled by the fluid between them and viscoelastic connectors. The calculated value of the gating-spring stiffness, determined by fitting mathematical models to experimental data, depends greatly on whether the OHB’s 3D morphology is taken into account (e.g., 8 mN/m using the 3D morphology versus 3.7 mN/m neglecting 3D morphology)^9,10^. This dependence suggests that the effects of gating-spring stiffness depend on the OHB’s 3D morphology. Here, we use mathematical models with and without the OHB’s 3D morphology to determine how gating-spring stiffness controls the mechanics and receptor current of OHBs.

## Results

### Gating-spring mutant OHB models

To investigate how gating-spring stiffness affects OHB responses to stimulation, we use a recently-published experimentally-constrained mathematical model of the OHB^10^. We call the OHB model with the default parameter values, the wild-type OHB. Parameters of the wild-type OHB correspond to OHBs in rats and mice from the 4-kHz characteristic-frequency region in the ear. Stereocilium columns are numbered relative to the central column, forming the left (negative numbers) and right (positive numbers) wings of the OHB (Fig. 1b). The wild-type OHB comprises viscoelastic pivots and connectors, fluid damping between neighboring stereocilia, two-state MET-channel kinetics, and MET channels gated by gating-springs. We vary the gating-spring stiffness in the wild-type OHB and call the resulting models mutant gating-spring OHBs.

Here, we focus on the question, for a fixed OHB morphology and resting (no stimulus) state (i.e., for a fixed geometry), how does gating-spring stiffness affect OHB responses to stimulus forces. In principle, changing the gating-spring stiffness changes the resting state of the OHB (Methods and Supplementary Information). We fix the resting state of the OHB when the gating-spring stiffness is changed by adjusting the unloaded length of the gating spring (length of the gating spring when the gating-spring tension is zero). All other parameter values in the mutant gating-spring OHBs equal those in the wild-type OHB.

The OHB is a 3D V-shaped structure, with a notch near the point of the V shape (Fig. 1). We define the X direction to be toward row 1, parallel to the column at the notch (column 0). The Y direction is toward the left wing of the OHB and perpendicular to the X direction, such that the XY plane defines the hair-cell apex. The Z direction is toward the tips of the stereocilia and perpendicular to the the hair-cell apex.

### Gating-spring stiffness increases Y deflections in response to an X-direction stimulus

The value of the gating-spring stiffness in the wild-type OHB (8 mN/m) is about an order of magnitude larger than the gating-spring stiffness of the well-characterized bundles in the frog sacculus (0.6 mN/m)^8–10^. Motivated by this observation, we first use a mutant gating-spring OHB model with a gating-spring stiffness that is one tenth of the wild-type value (0.8 mN/m), which we call the weak gating-spring mutant OHB.

*In vivo*, the tectorial membrane applies stimulus forces on the tips of the row-1 stereocilia^1,13^. The exact distribution of these forces is unknown, but they likely have similar timing and magnitude^10^. For identical stimulus forces on the row-1 tips, stimuli in the X direction maximize the receptor current^10^. Correspondingly, we apply identical stimulus forces in the X direction.

**Fig. 2:**
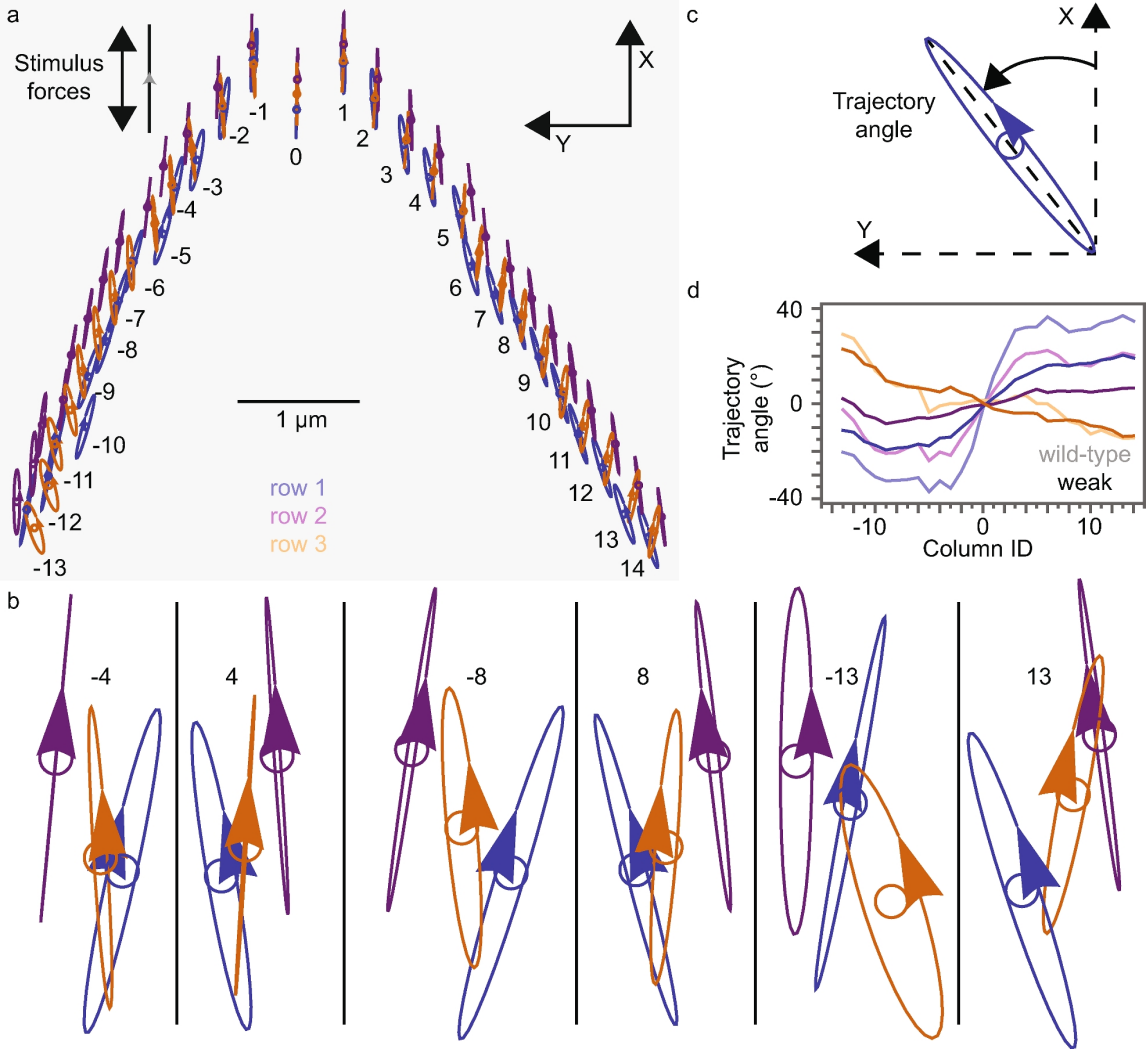
OHB stereocilia rotate in 3D owing to small characteristic-frequency (4 kHz) sinusoidal stimulus forces in the X direction (double-headed arrow). (**a**) Weak gating-spring mutant OHB stereocilium tips rotate (ellipse-like trajectories) around their resting positions (small circles). The resting positions and 3D trajectories are shown projected onto the hair-cell apex (XY plane). The scale bar applies only to the resting positions. Trajectories have been normalized to stereocilium heights. The arrow on each trajectory indicates the stereocilium displacement when the stimulus forces are at a maximum in the X direction (gray arrowhead on black line). (**b**) Magnified views are shown for trajectories illustrated in (a) corresponding to columns $-4$, 4, $-8$, 8, $-13$, and 13. (**c**) The definition of a trajectory’s angle in the XY plane is shown. (**d**) Trajectory angles are shown versus the column ID number corresponding to the wild-type (light colors) and the weak gating-spring mutant (dark colors) OHB. (All panels) Colors correspond to row 1 (blue), 2 (purple), and 3 (orange).

In response to small (1 pN amplitude per column; 28 pN total) characteristic-frequency sinusoidal forces applied in the X direction on the row-1 stereocilium tips, the stereocilium tips of the weak gating-spring mutant OHB rotate around their resting positions, forming ellipse-like trajectories (Fig. 2a). Like the wild-type OHB, the orientation angle of the tip trajectories generally increases with distance from column 0, the trajectories have opposite orientations in the left and right wings, and the row-3 trajectories have oppositely-signed orientations to those of rows 1 and 2 (Figs. 2 and S1)^10^. For the weak gating-spring mutant in comparison to the wild-type OHB, the row 2 and 3 trajectory angles are smaller in magnitude, such that there is less change between columns and smaller differences between rows. Larger trajectory angles in the wild-type OHB correspond to increased Y deflections relative to X deflections.

**Fig. 3:**
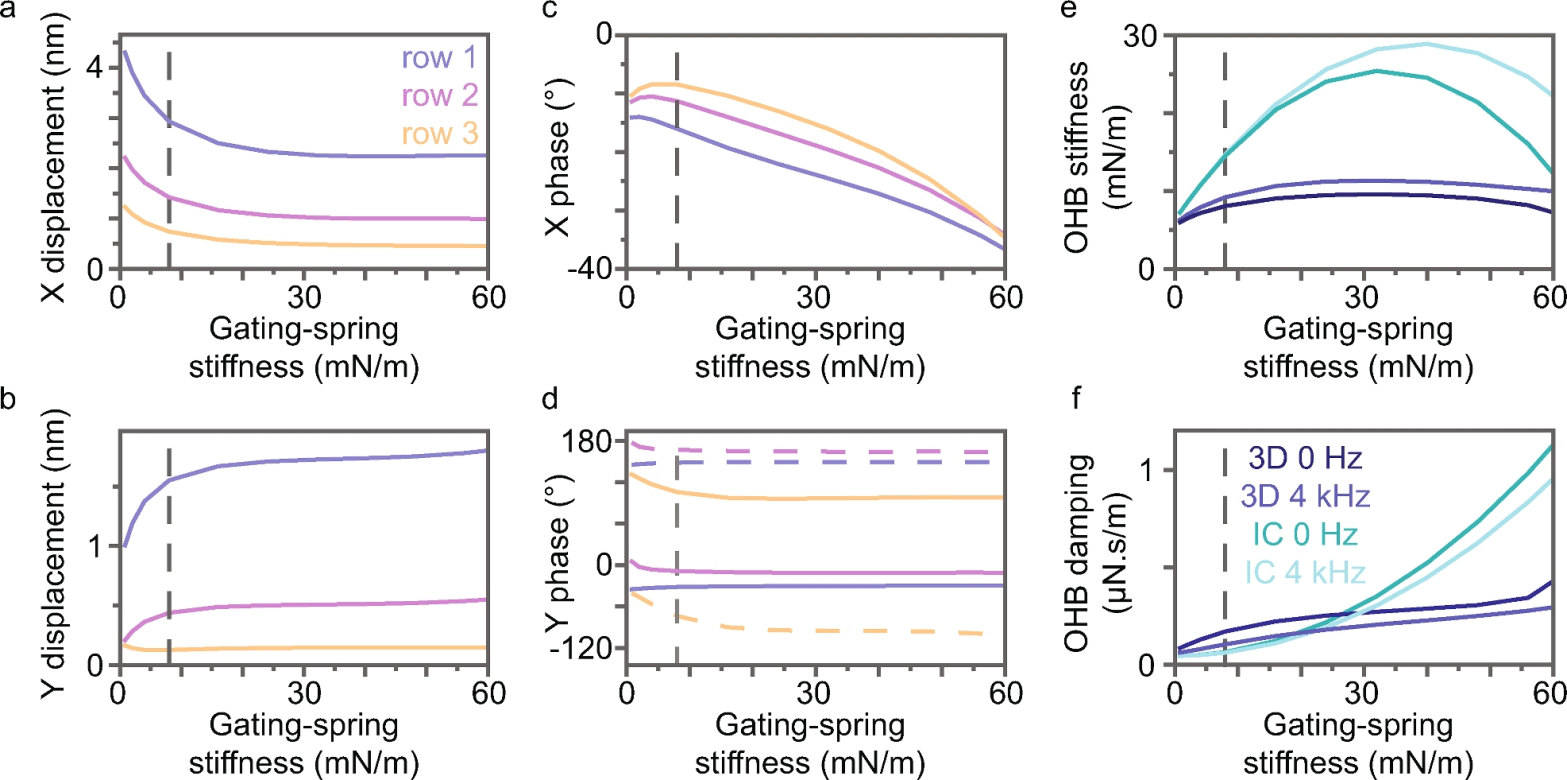
Characteristic-frequency deflections, stiffness, and damping depend on the gating-spring stiffness. (**a,b**) For each row, the mean displacement amplitudes in the X and Y directions are shown versus the gating-spring stiffness. (**c,d**) Phases of the mean X- and Y-displacements are shown versus the gating-spring stiffness. (**d**) Phases for left (dashed lines) and right (solid lines) wings are shown separately. (**a–d**) Colors correspond to row 1 (blue), 2 (purple), and 3 (orange). (**e,f**) The stiffness and damping coefficient associated with the mean row-1 displacement are shown versus the gating-spring stiffness for the 3D OHB stimulated at 0 Hz (dark blue, 3D 0 Hz), the 3D OHB stimulated at the characteristic frequency (light blue, 3D 4 kHz), the identical-columns OHB stimulated at 0 Hz (dark cyan, IC 0 Hz), and the identical-columns OHB stimulated at the characteristic frequency (light cyan, IC 4 kHz). (All panels) The wild-type gating-spring stiffness is indicated by dashed gray lines.

At the characteristic frequency, the mean stereocilium-tip displacement amplitude for each row and the phase of the mean displacement for each row change substantially when the gating-spring stiffness is changed (Fig. 3a–d). Although X and row-3 Y displacement amplitudes decrease with gating-spring stiffness, row-1 and row-2 Y amplitudes increase (Fig. 3a,b). X phases generally decrease with gating-spring stiffness, but Y phases increase (row 1) or decrease (rows 2 and 3) (Fig. 3c,d). Like the wild-type OHB, the left and right wings of the gating-spring mutant OHB move in antiphase^10^.

### Gating-spring stiffness increases OHB stiffness and damping

The stiffness and damping coefficient of the OHB are defined in terms of the mean row-1 X displacement^10^. For a 0-Hz stimulus, OHB stiffness peaks versus gating-spring stiffness (Fig. 3e). Considering a single gating spring attached to a single MET channel (a single gating-spring complex) provides insight into the decrease in OHB stiffness. The effective stiffness of a single gating-spring complex peaks versus gating-spring stiffness (Equation 15 in the Supplementary Information). The decrease is caused by MET-channel gating, an effect called gating compliance^7^. For a characteristic-frequency stimulus, OHB stiffness is larger and peaks versus gating-spring stiffness (Fig. 3e).

To understand how 3D morphology affects the OHB’s dependence on gating-spring stiffness, we use an identical-columns OHB, which lacks the 3D morphology^10^. The identical-columns OHB has the same parameters as the 3D OHB, except that its pivot positions are moved to make the columns identical (the row 1-2 and row 2-3 pivot spacings equal the average row 1-2 and row 2-3 pivot spacings in the 3D OHB) and fluid-coupling damping between columns and connectors between columns are removed to make the columns independent. Like the effective stiffness of a single gating-spring complex, the stiffness of the identical-columns OHB changes substantially with gating-spring stiffness and is a quadratic function of the gating-spring stiffness owing to gating compliance, and the peak stiffness increases with stimulus frequency because gating-compliance decreases with stimulus frequency (Fig. 3e; Equation 15 in the Supplementary Information). In comparison, 3D-OHB stiffness changes much less with gating-spring stiffness and does not depend quadratically on gating-spring stiffness. That is, the 3D morphology of the OHB decreases the gating-spring and MET-channel gating contributions to OHB stiffness.

MET-channel gating contributes to bundle damping because gating causes effective gating-spring damping (Equation 16 in the Supplementary Information)^19^. The size of the gating-spring force needed to open and close the MET channel increases with frequency because channel gating dissipates more energy with increasing frequency. Bundle damping caused by MET-channel gating has been measured previously in frog saccular bundles^19^.

Like the effective damping coefficient of a single gating-spring complex, the identical-columns OHB damping coefficient increases quadratically with gating-spring stiffness and decreases with stimulus frequency (Fig. 3f). The quadratic dependence and the small OHB damping at a gating-spring stiffness of zero imply that identical-columns OHB damping is primarily caused by MET-channel gating. The 3D-OHB damping coefficient changes much less with gating-spring stiffness, implying that the 3D morphology of the OHB decreases the contribution of MET-channel gating to OHB damping. In agreement with prior work, gating damping contributes little to wild-type OHB damping^10^. However, for gating-spring stiffness values less than 15 mN/m, damping in the 3D OHB is larger than in the identical-columns OHB. The additional damping in the 3D OHB is caused by fluid-coupling and connector damping between stereocilia in different columns^10^. Increasing gating-spring stiffness, increases damping between stereocilia in 3D by increasing differences in stereocilium trajectory orientations and increasing row-1 and row-2 Y displacements, causing OHB damping to increase (Figs. 2d and 3b,f). Because neighboring stereocilia move more similarly with increasing stimulus frequency, decreasing energy dissipation caused by fluid coupling and connectors, OHB damping is smaller for a 4-kHz than a 0-Hz stimulus (Fig. 3f).

### Gating-spring stiffness greatly changes the OHB displacement frequency response

**Fig. 4:**
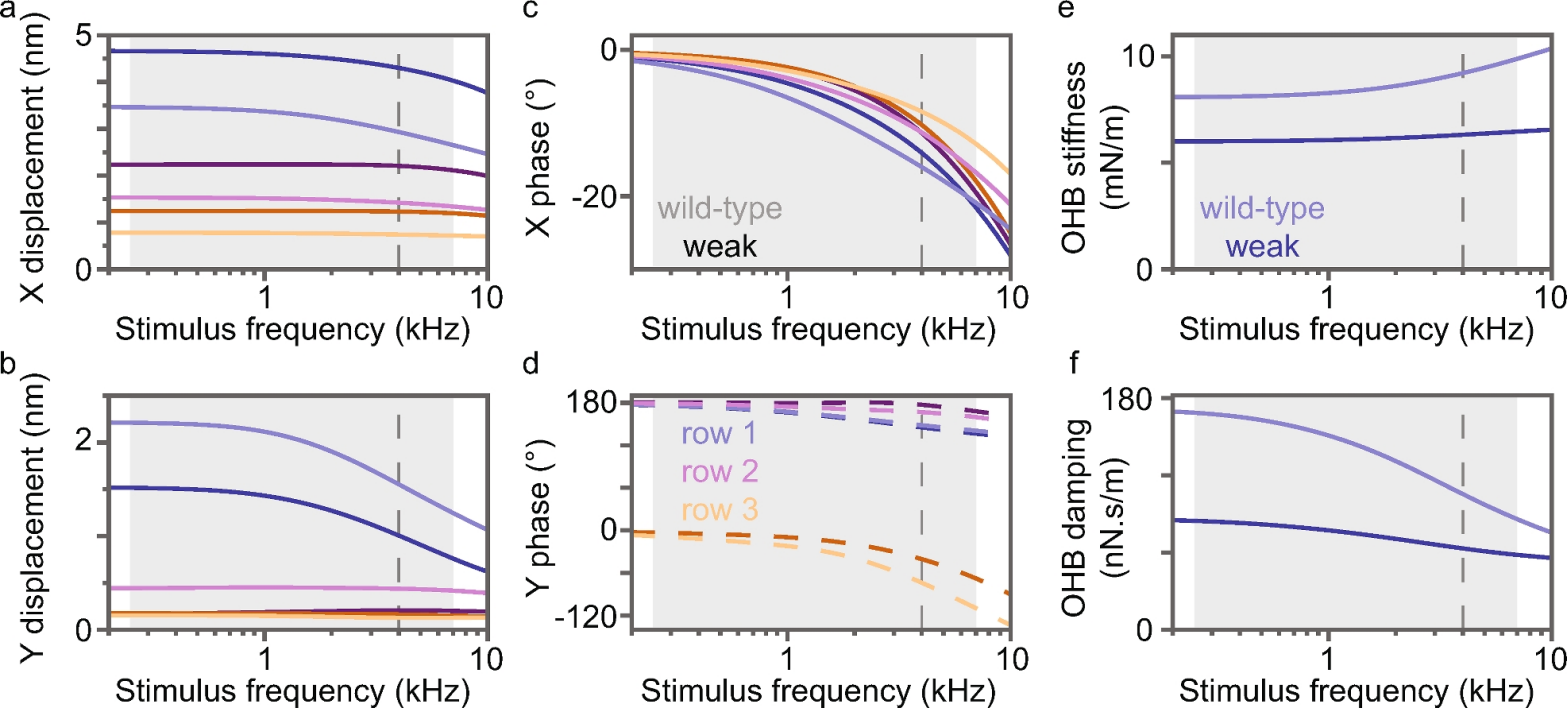
Displacements, stiffness, and damping versus stimulus frequency differ for wild-type (light lines) and weak gating-spring mutant (dark lines) OHBs. (**a,b**) The mean displacement amplitudes in the X and Y directions are shown versus the stimulus frequency. (**c,d**) Phases of the mean X- and Y-displacements are shown versus the stimulus frequency. (**d**) Phases of the mean Y-displacements for the left wing (dashed lines) are shown. (**e,f**) The stiffness and damping coefficient associated with the mean row-1 displacement are shown versus the stimulus frequency. (All panels) Colors correspond to row 1 (blue), 2 (purple), and 3 (orange), the characteristic frequency is indicated by dashed gray lines, and the physiological range of stimulus frequencies is indicated by gray boxes.

Because the physiological range of stimulus frequencies for an OHB at the 4-kHz characteristic-frequency place is about 250 Hz to 7 kHz, it is useful to consider responses to stimulus frequencies that encompass this range (Methods). The mean displacement amplitudes and phases of the mean displacements generally decrease with stimulus frequency in the weak gating-spring mutant and wild-type OHBs (Fig. 4a–d). Weak gating-spring mutant OHB X amplitudes are larger than those in the wild-type OHB, but Y amplitudes are smaller (rows 1 and 2) or of similar size (row 3) (Fig. 4a,b). X phases differ less between rows in the weak gating-spring mutant than in the wild-type OHB (Fig. 4c). Based on their Y phases, the left and right wings move in approximate antiphase at every stimulus frequency in the weak gating-spring mutant and wild-type OHBs. Row-2 and row-3 Y phases of the gating-spring mutant OHB are larger than those in the wild-type OHB (Fig. 4d).

OHB stiffness increases with stimulus frequency for both the wild-type and weak gating-spring mutant OHB (Fig. 4e). Weak gating-spring mutant OHB stiffness is smaller than in the wild-type OHB. The stiffness difference between the wild-type and weak gating-spring mutant OHB increases with stimulus frequency, implying that gating-spring stiffness contributes to the increase in OHB stiffness with increasing stimulus frequency.

OHB damping is smaller in the weak gating-spring mutant OHB than in the wild-type OHB (Fig. 4f). In the weak gating-spring mutant and wild-type OHBs, damping forces between neighboring stereocilia decrease their relative motions with increasing stimulus frequency, decreasing energy dissipation associated with these relative motions, which decreases OHB damping. The damping difference between the wild-type and weak gating-spring mutant OHB decreases with stimulus frequency, implying that gating-spring stiffness contributes less to OHB damping with increasing stimulus frequency (stereocilium displacements are constrained more by damping forces than by stiffness forces with increasing stimulus frequency).

### Gating-spring stiffness increases the receptor-current amplitude and phase

**Fig. 5:**
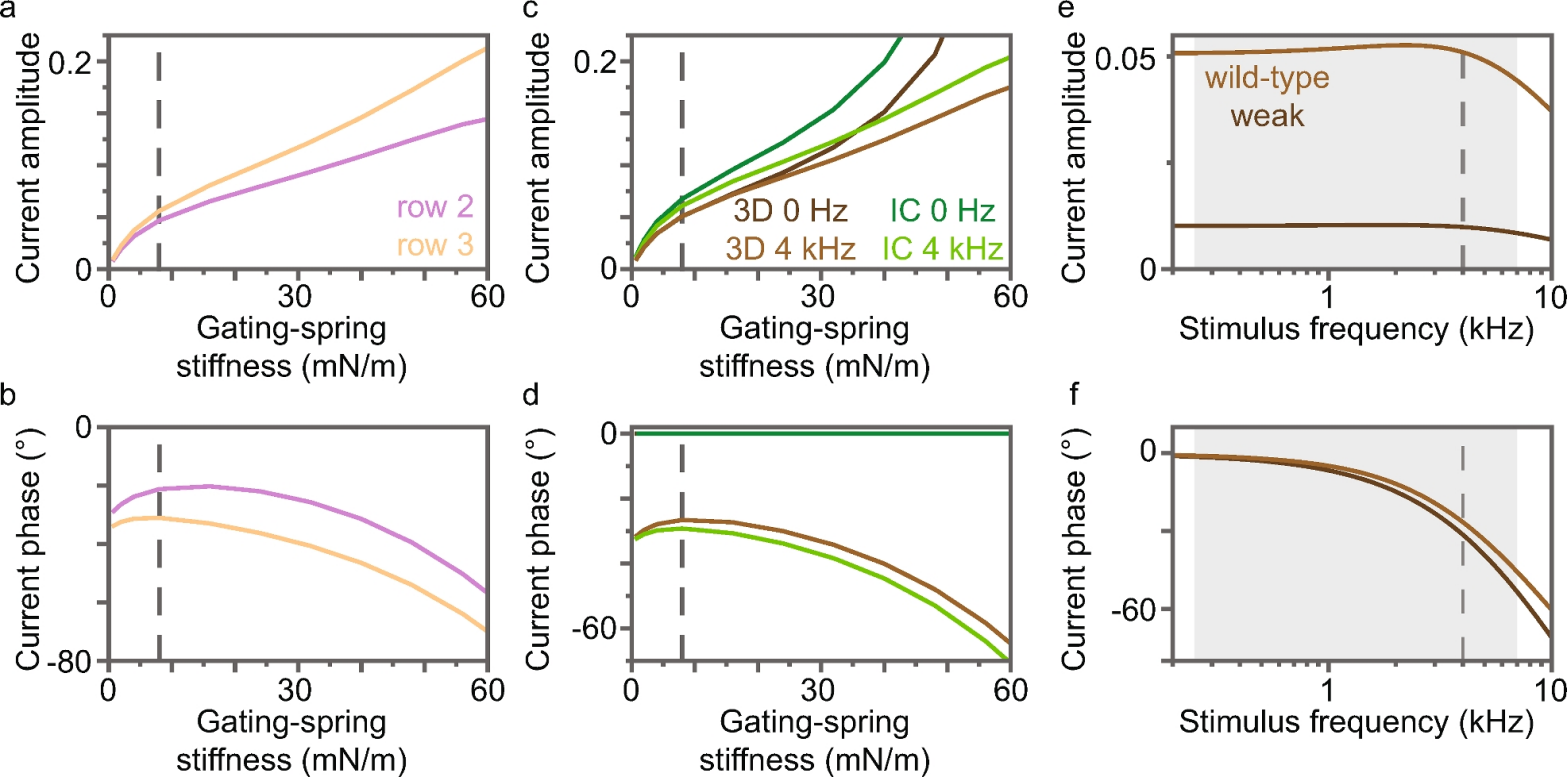
Normalized MET-channel currents depend on gating-spring stiffness. (**a,b**) Characteristic-frequency current amplitudes and phases are shown versus the gating-spring stiffness for row 2 (purple) and 3 (orange). (**c,d**) Receptor-current amplitudes and phases are shown versus the gating-spring stiffness for the 3D OHB stimulated at 0 Hz (dark brown, 3D 0 Hz), the 3D OHB stimulated at the characteristic frequency (light brown, 3D 4 kHz), the identical-columns OHB stimulated at 0 Hz (dark green, IC 0 Hz), and the 3D OHB stimulated at the characteristic frequency (light green, IC 4 kHz). The phases are zero for 0-Hz stimulation. (**a–d**) The wild-type gating-spring stiffness is indicated by dashed gray lines. (**e,f**) The receptor-current amplitude and phase are shown versus the stimulus frequency for the wild-type (light brown) and weak gating-spring mutant (dark brown) OHBs. The characteristic frequency is indicated by dashed gray lines and the physiological range of stimulus frequencies is indicated by gray boxes.

Channel current amplitudes (normalized to their maximum value) at the characteristic frequency increase and differ more between rows with increasing gating-spring stiffness (Fig. 5a). Current phases peak versus gating-spring stiffness and differ between rows (Fig. 5b). Like the current of a single gating-spring complex, receptor-current amplitudes (normalized) increase with gating-spring stiffness more rapidly for 0-Hz than for characteristic-frequency stimuli (Fig. 5c,d; Eq. 19 in the Supplementary Information). The receptor-current phase relative to the stimulus force peaks near the wild-type gating-spring stiffness and then decreases with increasing gating-spring stiffness and can become less than $-90^{\circ}$. In contrast, the phase of the current in a single gating-spring complex relative to the tension of its gating spring does not peak and is always greater than $-90^{\circ}$, implying that stereocilium phases contribute substantially to receptor-current phases in the wild-type and weak-gating spring mutant OHBs (Equation 21 in the Supplementary Information).

Receptor-current amplitudes in the identical-columns and 3D OHBs have similar dependencies on gating-spring stiffness (Fig. 5c,d). At each gating-spring stiffness, the 3D morphology of the OHB decreases the amplitudes and slightly increases the characteristic-frequency phase. Like the wild-type OHB, current amplitudes and phases in the weak gating-spring mutant OHB generally decrease with frequency (Fig. 5e,f). In the physiological stimulus frequency range, the weak gating-spring mutant OHB receptor-current amplitude and phase are smaller than those of the wild-type OHB. The amplitude difference decreases and the phase difference increases with stimulus frequency.

### Gating-spring stiffness decreases displacement-current and force-current dynamic ranges

**Fig. 6:**
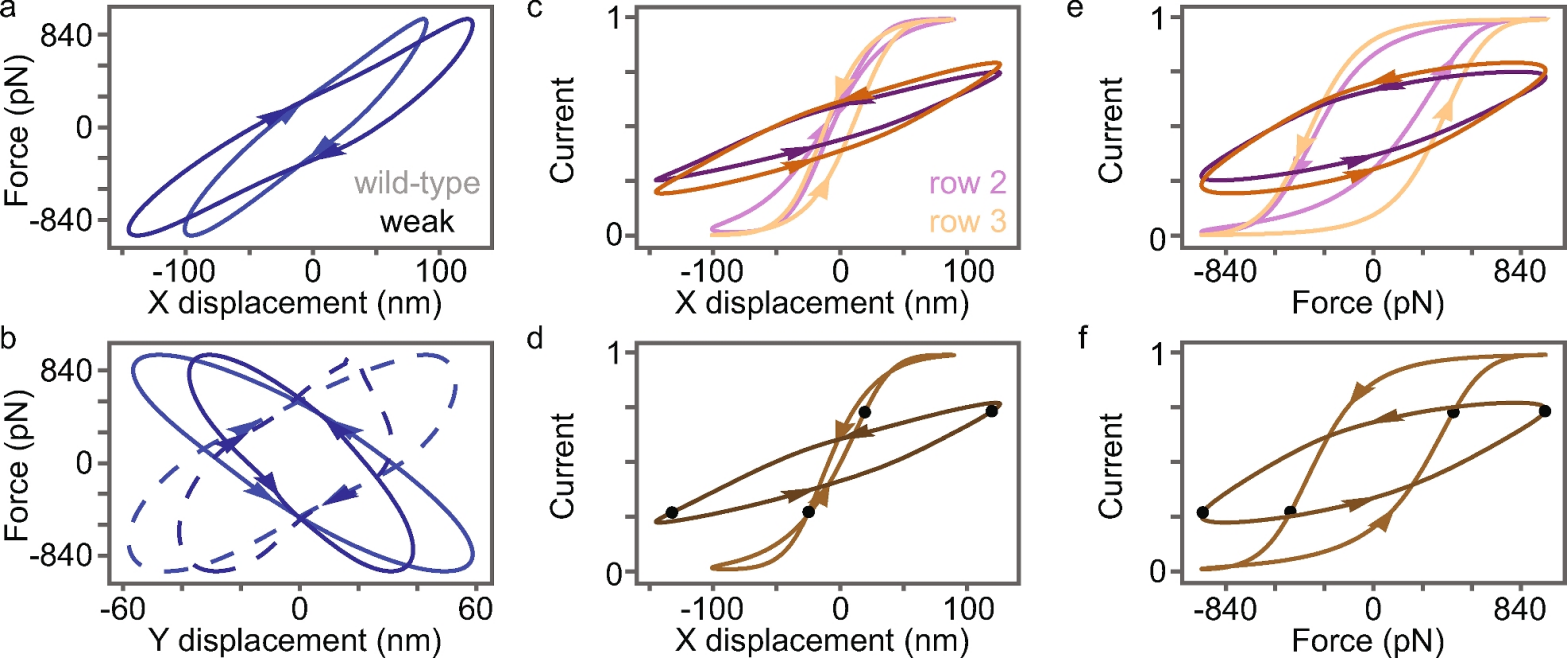
Characteristic-frequency stimulus displacement-force, displacement-current, and force-current relationships depend on gating-spring stiffness. (**a,b**) The force applied to the OHB is shown versus the mean row-1 X and Y displacement. (**b**) Curves for left (dashed lines) and right (solid lines) wings are shown separately. (**c,d**) The row-2 (purple), row-3 (orange), and receptor (brown) current are shown versus the mean row-1 X displacement. (**e,f**) The row-2 (purple), row-3 (orange), and receptor (brown) current are shown versus the stimulus force. (**d,f**) Black dots indicate the 0.27–0.73 dynamic ranges. (**c–f**) Currents are normalized by the maximum current. (All panels) Color shade indicates the wild-type (light lines) and weak gating-spring mutant (dark lines) OHBs. Arrows indicate how quantities change with time.

Next we examined nonlinear responses to characteristic-frequency stimulus forces (35 pN amplitude per column; 980 pN total) that almost completely open and close the wild-type OHB channels. X displacements are larger and Y displacements are smaller for the weak gating-spring mutant OHB (Fig. 6a,b). Like the small-stimulus responses, the wild-type OHB has a larger stiffness (minimum-to-maximum X slope) than the weak gating-spring mutant. The wild-type and weak gating-spring mutant OHB displacement-force relationships exhibit hysteresis (difference in the forward and backward branches of the response) for X and Y row-1 displacements, owing to damping and variation in row-1 stereocilium displacements. Decreasing gating-spring stiffness decreases displacement-force X hysteresis, consistent with decreasing X-displacement damping. In both the weak gating-spring mutant and wild-type OHBs, left and right wings move in Y in antiphase (Fig. 6b).

At most instantaneous displacements, row-2 and row-3 currents differ from each other less in the weak gating-spring mutant OHB than in the wild-type OHB (Fig. 6c). Unlike the wild-type OHB, row-2, row-3, and receptor currents in the weak gating-spring mutant OHB do not saturate (Fig. 6c,d). Correspondingly, the 0.27–0.73 displacement-current dynamic range of the wild-type OHB (44 nm) is almost six times smaller than that of the weak gating-spring mutant OHB (252 nm). However, a decrease by a factor of ten is expected considering a single gating-spring complex (Equation 2 and text in the Supplementary Information). The OHB’s 3D morphology decreases the displacement-current dynamic-range dependence on gating-spring stiffness. There is less displacement-current hysteresis in the wild-type than in the weak gating-spring mutant OHB. Additional variation in stereocilium displacements and currents in the wild-type OHB causes additional variation in the displacement-current curves for each MET channel, which decreases hysteresis in the mean currents of row 2, row 3, and the receptor current^10^.

Row-2 and row-3 currents differ from each other less in the weak gating-spring mutant OHB than in the wild-type OHB at most instantaneous stimulus forces (Fig. 6e). Unlike the wild-type OHB, row-2, row-3, and receptor currents in the weak gating-spring mutant OHB do not saturate for the forcing amplitude that saturates the wild-type OHB current (Fig. 6e,f). For small stimulus forces, there is more force-current hysteresis in the wild-type OHBs than in the weak gating-spring mutant OHB. The 0.27–0.73 force-current dynamic range of the weak gating-spring mutant OHB (1949 pN) is twice that of the wild-type OHB (927 pN). In contrast, the force-current dynamic range of a single gating-spring complex changes little with gating-spring stiffness (Equation 22 and text in the Supplementary Information). Therefore, the increase in the force-current dynamic range of the weak gating-spring mutant OHB must be caused by changes in 3D stereocilium displacements that drive the receptor current (Fig. 6a,b).

### Gating-spring stiffness increases receptor-current distortion products

In response to a stimulus force with two primary frequencies (3.2 kHz and 4 kHz; 490 pN total force for each primary), the OHB receptor current displays distortion products (Fig. S2). These distortion products are caused by dynamic-range nonlinearities (Fig. 6d,f). Because the wild-type OHB has a smaller dynamic range than the weak gating-spring mutant, the wild-type distortion products are 4.5-5.1 times larger (relative to the primaries) than those of the weak gating-spring mutant.

## Discussion

By comparing the weak gating-spring mutant OHB and the wild-type OHB, we find that the increased OHB gating-spring stiffness increases OHB stiffness (Figs. 3e and 4e), damping (Figs. 3f and 4f), receptor-current amplitudes (Fig. 5c,e), and receptor-current phases (Fig. 5d,f). Increasing the gating-spring stiffness above the wild-type OHB stiffness further increases OHB stiffness (up to a gating-spring stiffness of about 30 mN/m; Fig. 3e), damping (Fig. 3f), and receptor-current amplitudes (Fig. 5c), but decreases receptor-current phases (Fig. 5d). Additionally, the increased wild-type gating-spring stiffness decreases the displacement-current dynamic range (Fig. 6d), displacement-current hysteresis (Fig. 6d), and the force-current dynamic range (Fig. 6f), but increases force-current hysteresis (Fig. 6f).

MET-channel gating and the OHB’s 3D morphology affect how OHB responses depend on gating-spring stiffness. Neglecting the OHB’s 3D morphology, OHB stiffness and damping are predicted to change substantially with gating-spring stiffness and MET-channel gating (Fig. 3e,f). The OHB’s 3D morphology decreases the dependence of OHB stiffness and damping on gating-spring stiffness and MET-channel gating. OHB stiffness is smaller in the 3D OHB than in the identical-columns OHB in part because gating-spring stiffness contributes less to OHB stiffness in 3D. Below a gating-spring stiffness of 15 mN/m, OHB damping is larger in the 3D OHB than in the identical-columns OHB, because the OHB’s 3D morphology causes additional damping. The additional OHB damping is caused by connector and fluid-coupling damping associated with neighboring stereocilia in different columns.

In contrast to OHB stiffness and damping, gating-spring stiffness affects small-stimulus receptor currents similarly in the 3D and identical-columns OHBs (Fig. 5c,d). The rapid increase in the receptor current with gating-spring stiffness is caused by the relationship between MET-channel gating and gating-spring stiffness. Receptor-current phases are caused by stereocilium, gating-spring, and MET-channel phase differences. Overall, receptor-current amplitude depends much more on gating-spring stiffness than receptor-current phase (Fig. 5c–f).

For a large stimulus, displacement-current dynamic range depends more on gating-spring stiffness than displacement-current hysteresis, which increases with receptor-current phase lag (Fig. 6d). However, the OHB’s 3D morphology decreases the displacement-current dynamic range and hysteresis dependence on gating-spring stiffness substantially. The expected changes based on a single gating-spring complex are much larger than the changes seen in the 3D OHB. In contrast, a single-gating spring complex predicts little change in the force-current activation curves, but we find large changes in the 3D OHB (Fig. 6f). We conclude that the decrease in the force-current dynamic range and hysteresis with increasing gating-spring stiffness are caused by the OHB’s 3D morphology.

OHB stiffness increases toward the end of the cochlea that best responds to high-frequency sounds^9^. This stiffness increase is expected to increase the characteristic frequency of each cochlear location and to sharpen frequency tuning^14–17^. Correspondingly, the measured increase in gating-spring stiffness might increase OHB stiffness so as to increase the characteristic frequency and frequency tuning^9^. However, we find that gating-spring stiffness also increases OHB damping, which is expected to decrease the characteristic frequency and frequency tuning. To increase OHB displacements in response to near characteristic-frequency sounds *in vivo*, there may be trade-offs between the stiffness increase and damping increase owing to gating-spring stiffness.

In addition to the OHB receptor-current amplitude, cochlear amplification depends on phase differences between cochlear structures, OHB stimulus forces, OHB displacements, outer-hair-cell receptor potentials, and electromotility^15,16,20,21^. We find that gating-spring stiffness regulates the phase difference between OHB receptor currents and stimulus forces (Fig. 5d,f). Structures loading the OHB, like the tectorial membrane, may cause different stimulus forces on each row-1 stereocilium and bias the OHB’s resting position, which might change the receptor-current amplitude and phase. However, there is no evidence for stimulus-force differences on the scale of the OHB’s width and there is evidence that cochlear structures do not bias the OHB’s resting position^10,22^. Our results suggest that gating-spring stiffness regulates cochlear amplification by controlling receptor-current amplitude and phase.

Even though gating-spring stiffness increases OHB stiffness and damping, decreasing stereocilium displacements, gating-spring stiffness increases the receptor current. Essentially, gating-spring stiffness has opposing effects on the transmission of stimulus energy to stereocilium displacements and the transmission of energy from stereocilium displacements to MET-channel gating. Gating-spring stiffness may be tuned in the cochlea to increase the stimulus energy delivered to the OHB, while ensuring a large receptor current for a given OHB displacement.

Gating-spring stiffness and MET-channel gating may have larger effects on bundle stiffness and damping in frog saccular bundles than in the OHBs studied here, because they differ greatly in morphology and mechanics^7,8,19^. Frog saccular bundles have over seven rows of stereocilia, are taller (8.45 $\mu$m), have smaller height differences between rows (325 nm), have wider stereocilia (380 nm), have larger separations between stereocilia (780 nm between pivot positions), and have stereocilia arranged in a circular pattern, whereas the OHBs described here have three rows of stereocilia, are shorter (4.1 $\mu$m), have larger height differences between rows (800–1000 nm), have narrower stereocilia (250 nm), have smaller separations between stereocilia (381 nm between pivot positions within rows; 603 nm between pivot positions within columns) and have stereocilia arranged in a V pattern^10,23^. Differences in mechanics include: pivot stiffness is 100 times smaller, gating-swing is 10 times larger, and the MET-channel timescale is 29 times longer in frog saccular bundles than in OHBs^10^. Finally, MET-channel gating causes less gating compliance and damping with increasing stimulus frequency, implying that MET-channel gating may have larger effects on bundle stiffness and damping at the lower stimulus frequencies (15–200 Hz) detected by frog saccular bundles (Equations 15 and 16 in the Supplementary Information)^24^. Inner-hair-cell bundles differ substantially in morphology and mechanics from OHBs, but we need more accurate and precise information about their 3D morphology and mechanics to determine whether gating-spring stiffness has large or small effects on inner-hair-cell bundle stiffness and damping^25–27^.

In response to a step stimulus, the receptor current increases, peaks, and then decreases. This decrease is known as adaptation^1^. The mechanisms underlying adaptation in OHBs are unclear^4^. For example, there is conflicting experimental data on whether adaptation depends on calcium influx through the MET channels. Because of the many uncertainties associated with OHB adaptation, we choose to omit adaptation in the OHB models presented here. We expect adaptation to modify the results for stimulus frequencies below 1 kHz, because the fastest adaptation rate measured in OHBs is less than 1 kHz^28^. How gating-spring stiffness affects adaptation depends on the adaptation mechanism. Adaptation likely high-pass filters the receptor current and may cause active hair-bundle motions like spontaneous hair-bundle oscillations^15,16,20,29,30^. However, these effects have not yet been seen in OHBs. Increasing gating-spring stiffness is expected to increase the high-pass filter corner frequency and bring the OHB closer to spontaneously oscillating^15,16,20,29,30^.

Frequency tuning in the cochlea may be caused by a combination of feedback between the mechanical and electrochemical domains, the inertia of large structures like the tectorial membrane, and the cochlear traveling wave^15,16,20,29,30^. We expect the weak frequency tuning we find in the OHB receptor current to contribute little to frequency tuning in the cochlea (Figs. 4a,b and 5e,f). However, the high-pass filter caused by adaptation combined with the low-pass filter we describe here is expected to cause sharper frequency tuning in the receptor current and can in principle cause frequency tuning of OHB displacements^15,16,20,29,30^.

The effects of gating-spring stiffness described here agree with several published experimental observations. OHB stiffness decreases when gating springs are broken in experiment, in agreement with the decrease in OHB stiffness when gating-spring stiffness is decreased (Figs. 3e and 4e)^9^. Variation in row-1 displacements decreases when gating springs are broken in experiment, in agreement with the decreased variation in row-1 displacements when gating-spring stiffness is decreased (Fig. 2)^31^. Decreased gating-spring stiffness in a mutant increases the displacement-current dynamic range in experiment, in agreement with the increased displacement-current dynamic range in the weakened gating-spring mutant OHB (Fig. 6d)^3^.

Receptor-current dynamic-range nonlinearities causes distortions in otoacoustic emissions (sounds driven by the cochlea) and in cochlear vibrations^15,32,33^. Distortion-product otoacoustic emissions are used to assess hearing loss associated with outer-hair-cell dysfunction in animals and humans^32–34^. We find that decreasing OHB gating-spring stiffness reduces receptor-current distortion products, implying that distortions in otoacoustic emissions and cochlear vibrations will be reduced in mutants with weak gating springs even if these mutants lack outer-hair-cell loss (e.g., LHFPL5 knockout mice)^3,34^. These reductions may be larger than we predict, because cochlear amplification increases distortion products but we use mathematical models lacking amplification.

Gating-spring stiffness may differ in different types of hair bundles owing to molecular differences in their tip links and other molecules in series with the MET channel^1,3,9,23^. However, tip links are polymers described by nonlinear force-extension curves^35,36^. Increasing the resting tension in such a polymer increases its stiffness^37^. OHBs have larger resting tensions (e.g., 34 pN at a 4-kHz characteristic frequency) than frog saccular bundles (8 pN) and inner-hair-cell bundles (e.g., 16 pN at a 4-kHz characteristic frequency) with the same characteristic frequency, which may contribute to the increased gating-spring stiffness in OHBs^8,9,38^. It is possible that resting tension tunes gating-spring stiffness in different types of hair bundles and in different locations with their sensory organs to improve hair-bundle responses to different types of sensory stimuli.

## Methods

### 3D OHB model

To determine how gating-spring stiffness regulates OHB responses in 3D, we use the 3D-OHB model described in prior work^10^. In this model, there are 56 gating springs, each gating an MET-channel. The receptor current equals the sum of the currents through the 56 channels. At rest, all gating lengths are the same, all resting gating-spring tensions are the same, and all MET channels have the same resting normalized MET current equalling 0.5. A resting normalized MET current of 0.5 maximizes the receptor-current sensitivity to deflections. For each pair of stereocilia in the same column, the gating length equals the stereocilium radius plus the tip-link length, which depends on the stereocilium deflections in 3D. Resting gating-spring tensions do not depend on the gating-spring stiffness, because myosin motors are assumed to set the resting gating-spring tensions.

We ensure that the mutant gating-spring OHBs have maximum receptor-current sensitivity by setting the resting normalized MET currents to 0.5. Because the gating-spring tensions, resting gating lengths, and resting MET currents are fixed, the gating-spring unloaded length must be adjusted as the gating-spring stiffness is varied. To understand, how and why the gating-spring unloaded length must be adjusted, we consider a single MET channel gated by a single gating spring (a single gating-spring complex) in the supplementary information.

Mathematical modeling is done using Mathematica 13.3.

### Physiological-frequency range

We estimate the physiological range of frequencies using data in mice and rats, because our mathematical models are based on mouse and rat data^10^. The 4-kHz characteristic-frequency place is stimulated by sound frequencies as low as the behaviorally-measured limit. This limit is about 800 Hz in mice and 250 Hz in rats^39–41^. For the the high-frequency limit, we consider the measured responses of auditory nerve fibers to 90-dB SPL stimuli. 5-kHz characteristic-frequency fibers respond to 7-kHz sounds in mice (a fractional difference of 2/5) and 11-kHz characteristic-frequency fibers respond to 18-kHz sounds in rats (a fractional difference of 7/11). Using the maximum fractional difference of 7/11, 4-kHz fibers will respond to 7-kHz sounds at 90 dB SPL^42,43^. To ensure we account for the full range, we estimate the physiological range for the 4-kHz characteristic-frequency place to be 250 Hz to 7 kHz.

## Supplementary Information

Supplementary Information.

## Data Availability

The code and data associated with the paper is available at Dryad: doi:10.5061/dryad.bnzs7h4kz.
