# Supplementary material for: Gating-spring stiffness increases outer-hair-cell bundle stiffness, damping, and receptor current
Source: Sci Rep. 2024 Dec 2;14:29904. doi: 10.1038/s41598-024-81355-5 (PMC11612202; doi:10.1038/s41598-024-81355-5)
Supplement: Supplementary file 1 — Supplementary Information. [file 41598_2024_81355_MOESM1_ESM.pdf]

Supporting Information for Gating-Spring Stiffness Increases Outer-Hair-Cell Bundle Stiffness, Damping, and Receptor Current

Zenghao Zhu[1], Wisam Reid[1,2], and Dáibhid Ó Maoiléidigh[1,*]

[1]Department of Otolaryngology-Head and Neck Surgery, Stanford University, Stanford, CA 94304, USA
[2]Harvard Medical School, Harvard University, Boston, MA 02114, USA
[*]dmelody@stanford.edu

Single gating-spring complex

In the 3D OHB, each MET channel in the OHB is described by an open and closed state[1]. The current through a channel normalized by the maximum current equals the open probability of the channel. For stimuli much slower than MET-channel opening and closing timescales, the quasistatic open probability of a channel is given by

$$P_\infty(g) = \left(1 + \exp\left(-\frac{k_\mathrm{g} d}{k_\mathrm{B} T}(g - g_\mathrm{h})\right)\right)^{-1}, \tag{1}$$

in which g is the gating length, k_g is the gating-spring stiffness, d is the gating swing, k_B is the Boltzmann constant, T is the temperature, and g_h is the half-activation extension ($P_\infty(g_\mathrm{h}) = 0.5$). The 0.27–0.73 dynamic range of the $P_\infty(g)$ versus g curve (the MET-channel activation curve) is given by 2δ, in which

$$\delta = \frac{k_\mathrm{B} T}{k_\mathrm{g} d}, \tag{2}$$

and the center is given by g_h. Decreasing k_g by a factor of 10 increases the 0.27–0.73 dynamic range by a factor of 10. The half-activation extension depends on the gating-spring stiffness according to

$$g_\mathrm{h} = \frac{k_\mathrm{B} T}{k_\mathrm{g} d} \ln\left(\frac{\kappa^-}{\kappa^+}\right) + \frac{G_\mathrm{ch}}{k_\mathrm{g} d} + g_\mathrm{u} + \frac{d}{2}, \tag{3}$$

in which κ^- and κ^+ are transition rate frequency factors, G_ch is the intrinsic energy difference between the MET channel's states (open energy minus closed energy), and g_u is the unloaded length of the gating spring.

The open probability of an MET channel at time t is given by

$$\tau \dot{P}_\mathrm{o}(t) = -P_\mathrm{o}(t) + P_\infty. \tag{4}$$

The gating timescale τ is a function of g given by

$$\tau(g) = \frac{P_\infty}{\kappa^+} \exp\left(-\frac{k_\mathrm{g} d}{k_\mathrm{B} T} \alpha g\right) \exp\left(\frac{G_\mathrm{b} + \alpha(G_\mathrm{ch} + k_\mathrm{g} d g_\mathrm{u} + k_\mathrm{g} d^2/2)}{k_\mathrm{B} T}\right), \tag{5}$$

in which α is a transition-state distance and G_b is the height of the energy barrier between the open and closed states.

The tension applied to the gating spring is given by

$$T_\mathrm{g} = k_\mathrm{g}(g - g_\mathrm{u} - d P_\mathrm{o}(t)), \tag{6}$$

such that the resting gating-spring tension is

$$T_\mathrm{g}^* = k_\mathrm{g}(g^* - g_\mathrm{u} - d P_\infty(g^*)). \tag{7}$$

The resting gating length $g^* = l_\mathrm{tl}^* + R$, in which l_tl^* is the resting tip-link length, R is the radius of a stereocilium, and the resting tension is independent of the gating-spring stiffness, because it is set by myosin motors. Equation 7 is a nonlinear equation in g^* with either one stable resting state or three resting states (two stable and one unstable). There is one stable resting state when $k_\mathrm{g} < k_\mathrm{gb}$, in which

$$k_\mathrm{gb} = \frac{k_\mathrm{B} T}{P_\infty^*(1 - P_\infty^*) d^2}. \tag{8}$$

At $k_\mathrm{g} = k_\mathrm{gb}$, there is a cusp bifurcation when $P^*_\infty = 0.5$ or a saddle-node bifurcation when $P^*_\infty \neq 0.5$[2]. A sufficiently large gating-spring stiffness is predicted to make a mechanically passive hair-bundle bistable, with either mostly closed or mostly open MET channels at rest. For the parameters of the 3D OHB ($d = 0.5$ nm, $T = 298$ K, and $P^*_\infty = 0.5$), $k_\mathrm{g} < 65$ mN/m ensures that there is one stable resting state.

According to equations 1–7, the gating-spring stiffness affects the dynamic range of the quasistatic open probability curve (2δ), the center of the quasistatic open probability curve (g_h), the resting gating timescale ($\tau(g^*)$), the resting gating length (g^*), and the resting open probability ($P_\infty(g^*)$). We are interested in determining how the gating-spring stiffness affects OHB function when the resting state is fixed to equal that of the wild-type OHB. Fixing the gating length and open probability causes the unloaded length to depend on the gating-spring stiffness (equation 7) according to

$$g_\mathrm{u} = g^* - \frac{T^*_\mathrm{g}}{k_\mathrm{g}} - dP_\infty(g^*) \tag{9}$$

and causes g_h, the resting gating timescale ($\tau(g^*)$), and the resting state of the OHB to be independent of the gating-spring stiffness. The dynamic range of the quasistatic open probability curve and the dynamic gating timescale, gating length, and open probability still depend on the gating-spring stiffness.

Effective stiffness and damping of a single gating-spring complex

We gain insight into how OHB stiffness and damping depend on the gating-spring stiffness by considering the effective stiffness and damping of a single gating-spring complex when the stimulus is small. To linear order around the resting state, equation 4 becomes

$$\tau^* \Delta\dot{P}_\mathrm{o} = -\Delta P_\mathrm{o} + (\partial_g P_\infty)^* \Delta g, \tag{10}$$

in which $\Delta P_\mathrm{o} = P_\mathrm{o} - P_\infty(g^*)$. For small sinusoidal gating-spring oscillations around the resting state at stimulus frequency ω, $\Delta P_\mathrm{o} = P^\mathrm{a}_\mathrm{o} \exp(i\omega t)$, in which P^a_o is a complex amplitude and we get

$$i\omega\tau^* \Delta P_\mathrm{o} = -\Delta P_\mathrm{o} + (\partial_g P_\infty)^* \Delta g, \tag{11}$$

yielding

$$\Delta P_\mathrm{o} = \frac{(\partial_g P_\infty)^*(1 - i\omega\tau^*)}{1 + (\omega\tau^*)^2} \Delta g. \tag{12}$$

Likewise, for small sinusoidal gating-spring oscillations around the resting state, the change in the gating-spring tension $\Delta T_\mathrm{g} = T_\mathrm{g} - T^*_\mathrm{g}$ becomes

$$\begin{aligned}
\Delta T_\mathrm{g} &= k_\mathrm{g}(\Delta g - d\Delta P_\mathrm{o}) \\
&= k_\mathrm{g}\left(1 - d\frac{(\partial_g P_\infty)^*(1 - i\omega\tau^*)}{1 + (\omega\tau^*)^2}\right)\Delta g \\
&= \left(\left(\frac{(\partial_g P_\infty)^* k_\mathrm{g} d\tau^*}{1 + (\omega\tau^*)^2}\right) i\omega + k_\mathrm{g}\left(1 - \frac{(\partial_g P_\infty)^* d}{1 + (\omega\tau^*)^2}\right)\right)\Delta g \\
&= \left(k_\mathrm{g}\left(1 - \frac{(\partial_g P_\infty)^* d}{1 + (\omega\tau^*)^2}\right) + i\omega\left(\frac{(\partial_g P_\infty)^* k_\mathrm{g} d\tau^*}{1 + (\omega\tau^*)^2}\right)\right)\Delta g \\
&\equiv (k_\mathrm{e} + i\omega\lambda_\mathrm{e})\Delta g,
\end{aligned} \tag{13}$$

in which k_e is an effective stiffness and λ_e is an effective damping coefficient. Equation 13 shows how MET-channel gating causes damping and stiffness forces.

The slope of the MET-channel activation curve or sensitivity of the open probability to small gating changes is

$$\partial_g P_\infty = \frac{k_\mathrm{g} d}{k_\mathrm{B} T} P_\infty (1 - P_\infty) \tag{14}$$

and is a maximum at $P_\infty = 0.5$. Fixing the resting open probability to be 0.5 ensures that the OHB model has maximum sensitivity for each gating-spring stiffness value.

The effective stiffness and damping coefficient can now be written as

$$k_\mathrm{e} = k_\mathrm{g}\left(1 - \frac{k_\mathrm{g} d^2}{k_\mathrm{B} T} \frac{P^*_\infty(1 - P^*_\infty)}{1 + (\omega\tau^*)^2}\right) \text{ and} \tag{15}$$

$$\lambda_e = \frac{k^2_\mathrm{g} d^2}{k_\mathrm{B} T} \frac{P^*_\infty(1 - P^*_\infty)}{1 + (\omega\tau^*)^2} \tau^*. \tag{16}$$

The effective stiffness depends quadratically on gating-spring stiffness, increasing and then decreasing with gating-spring stiffness, peaking at $k_{\text{gb}}/2$ (equation 8). MET-channel gating causes the decrease, an effect known as gating compliance[3]. Equation 15 shows that the contribution of MET-channel gating to the effective stiffness decreases with stimulus frequency. Note that there is a positive effective stiffness at rest (stimulus frequency of zero) if and only if there is one stable resting state (equation 8). MET-channel gating also causes damping[4]. Equation 16 shows that the effective damping depends quadratically on gating-spring stiffness and the contribution of MET-channel gating to the effective damping decreases with stimulus frequency. Equations 15 and 16 are extensions of prior expressions to the frequency-dependent cases[3,4].

Open probability, gating length, and gating-spring tension relationships

The change in the open probability (ΔP_{o}), the gating length (Δg), and the tension (ΔT_{g}) are related according to

$$\Delta P_{\text{o}} = \frac{k_{\text{g}}d}{k_{\text{B}}T}\frac{P_\infty^*(1-P_\infty^*)(1-i\omega\tau^*)}{1+(\omega\tau^*)^2}\Delta g \tag{17}$$

$$= \frac{k_{\text{g}}d}{k_{\text{B}}T}\frac{P_\infty^*(1-P_\infty^*)(1-i\omega\tau^*)}{1+(\omega\tau^*)^2}\frac{\Delta T_{\text{g}}}{\lambda_{\text{e}}i\omega+k_{\text{e}}} \tag{18}$$

$$= \frac{d}{k_{\text{B}}T}P_\infty^*(1-P_\infty^*)\left(1-\frac{k_{\text{g}}d^2}{k_{\text{B}}T}P_\infty^*(1-P_\infty^*)+i\omega\tau^*\right)^{-1}\Delta T_{\text{g}}. \tag{19}$$

For given stimulus tension amplitude ΔT_{g}, equation 19 implies that $|\Delta P_{\text{o}}|$ increases rapidly as k_{g} approaches k_{gb} and diverges at k_{gb} if the stimulus frequency is zero (equation 8). The open-probability change is low-pass filtered relative to the gating-extension change with a corner frequency of $1/(2\pi\tau^*)$ and relative to the tension change with a corner frequency of

$$\frac{1-P_\infty^*(1-P_\infty^*)k_{\text{g}}d^2/(k_{\text{B}}T)}{2\pi\tau^*}. \tag{20}$$

The corner frequency of the open-probability change relative to the tension change decreases with the gating-spring stiffness k_{g} and is positive when $k_{\text{g}} < k_{\text{gb}}$ (equation 8).

Equations 17 and 19 also imply that the change in the open probability is not in phase with the changes in gating length or the gating-spring tension. The phase of ΔP_{o} relative to ΔT_{g} (ψ_{PT}) is given by

$$\tan(\psi_{\text{PT}}) = \frac{-\omega\tau^*}{1-P_\infty^*(1-P_\infty^*)k_{\text{g}}d^2/(k_{\text{B}}T)}, \tag{21}$$

which decreases linearly with stimulus frequency. At a fixed stimulus frequency, the phase ψ_{PT} decreases rapidly to $-\pi/2$ as k_{g} increases to k_{gb} (equation 8).

For quasistatic stimuli, the difference between two gating-spring tension values is given by

$$T_{\text{g}1} - T_{\text{g}2} = \frac{k_{\text{B}}T}{d}\ln\left(\frac{P_{\infty2}^{-1}-1}{P_{\infty1}^{-1}-1}\right) - k_{\text{g}}d\left(P_{\infty1}-P_{\infty2}\right), \tag{22}$$

which equals the 0.27–0.73 dynamic range of the force-current activation curve when $P_{\infty1} = 0.73$ and $P_{\infty2} = 0.27$. The 0.27–0.73 dynamic range decreases very slowly with increasing k_{g}. Decreasing k_{g} by a factor of 10 decreases the 0.27–0.73 dynamic range by only a factor of 0.9.

When the stimulus is large, equations 4 and 6 can be numerically integrated. For sinusoidal tension stimuli at 4 kHz, there is little hysteresis in the resulting displacement-force curves, but there is considerable hysteresis in the displacement-current and force-current curves. Like the quasistatic case (equations 2 and 22), the dynamic range of the displacement-current curve is approximately inversely proportional to the gating-spring stiffness and the dynamic-range of the force-current curve changes very slowly with gating-spring stiffness.

References

1. Zhu, Z., Reid, W., George, S. S., Ou, V. & Ó Maoiléidigh, D. 3D morphology of an outer-hair-cell hair bundle increases its displacement and dynamic range. *Biophys J* **123**, 3433–3451, DOI: 10.1016/j.bpj.2024.08.009 (2024).
2. Ó Maoiléidigh, D., Nicola, E. M. & Hudspeth, A. J. The diverse effects of mechanical loading on active hair bundles. *Proc. Natl. Acad. Sci. USA* **109**, 1943–1948 (2012).
3. Howard, J. & Hudspeth, A. J. Compliance of the hair bundle associated with gating of mechanoelectrical transduction channels in the bullfrog's saccular hair cell. *Neuron* **1**, 189–199 (1988).
4. Bormuth, V., Barral, J., Joanny, J.-F., Jülicher, F. & Martin, P. Transduction channels' gating can control friction on vibrating hair-cell bundles in the ear. *Proc. Natl. Acad. Sci. USA* **111**, 7185–7190 (2014).

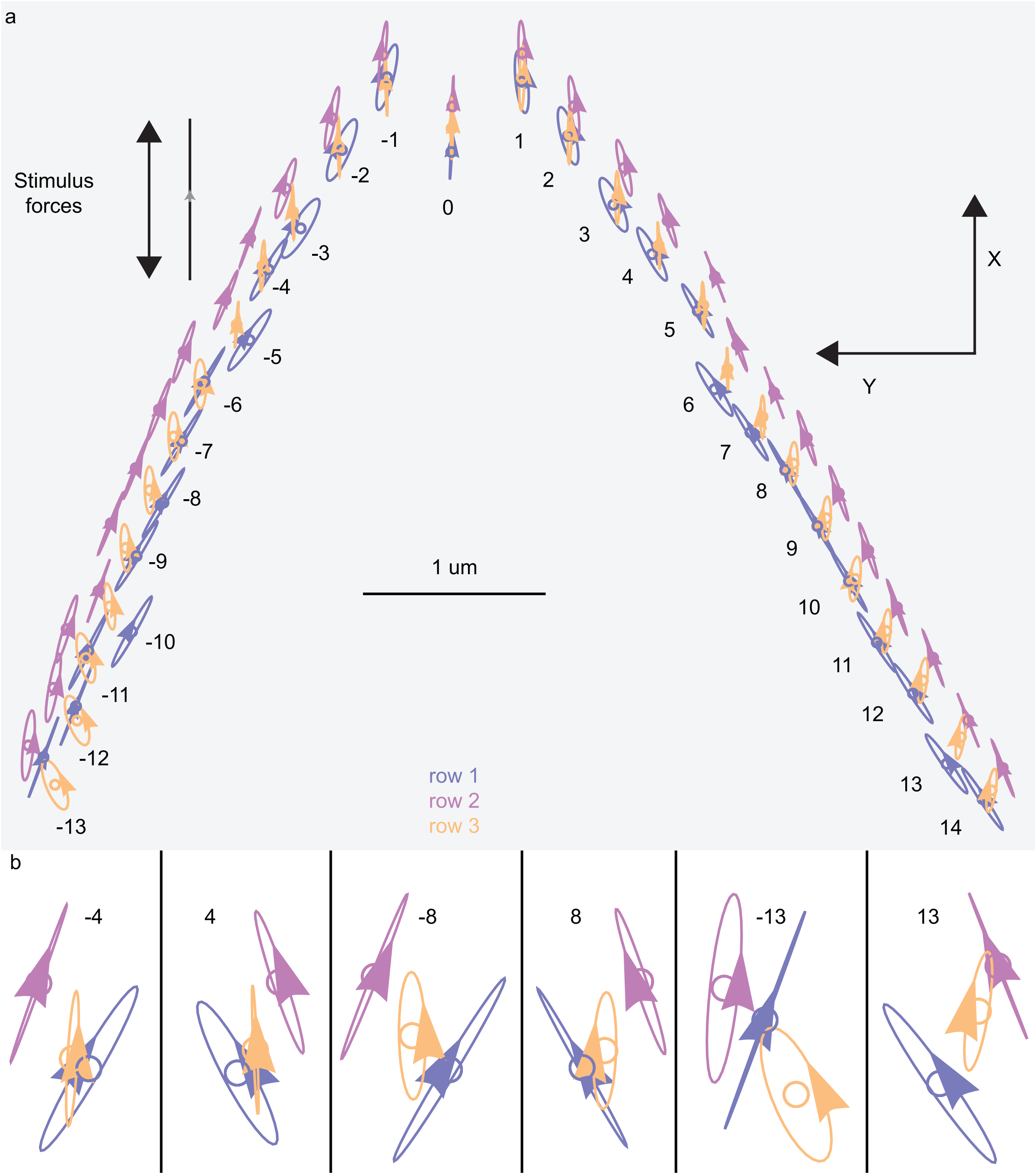

Figure S1. Wild-type OHB stereocilia rotate in 3D owing to small characteristic-frequency (4 kHz) sinusoidal stimulus forces in the X direction (double-headed arrow). (a) OHB stereocilium tips rotate (ellipse-like trajectories) around their resting positions (small circles). The resting positions and 3D trajectories are shown projected onto the hair-cell apex (XY plane). The scale bar applies only to the resting positions. Trajectories have been normalized to stereocilium heights. The arrow on each trajectory indicates the stereocilium displacement when the stimulus forces are at a maximum in the X direction (gray arrowhead on black line). (b) Magnified views are shown for some columns of the trajectories illustrated in (a). (All panels) Colors correspond to row 1 (blue), 2 (purple), and 3 (orange).

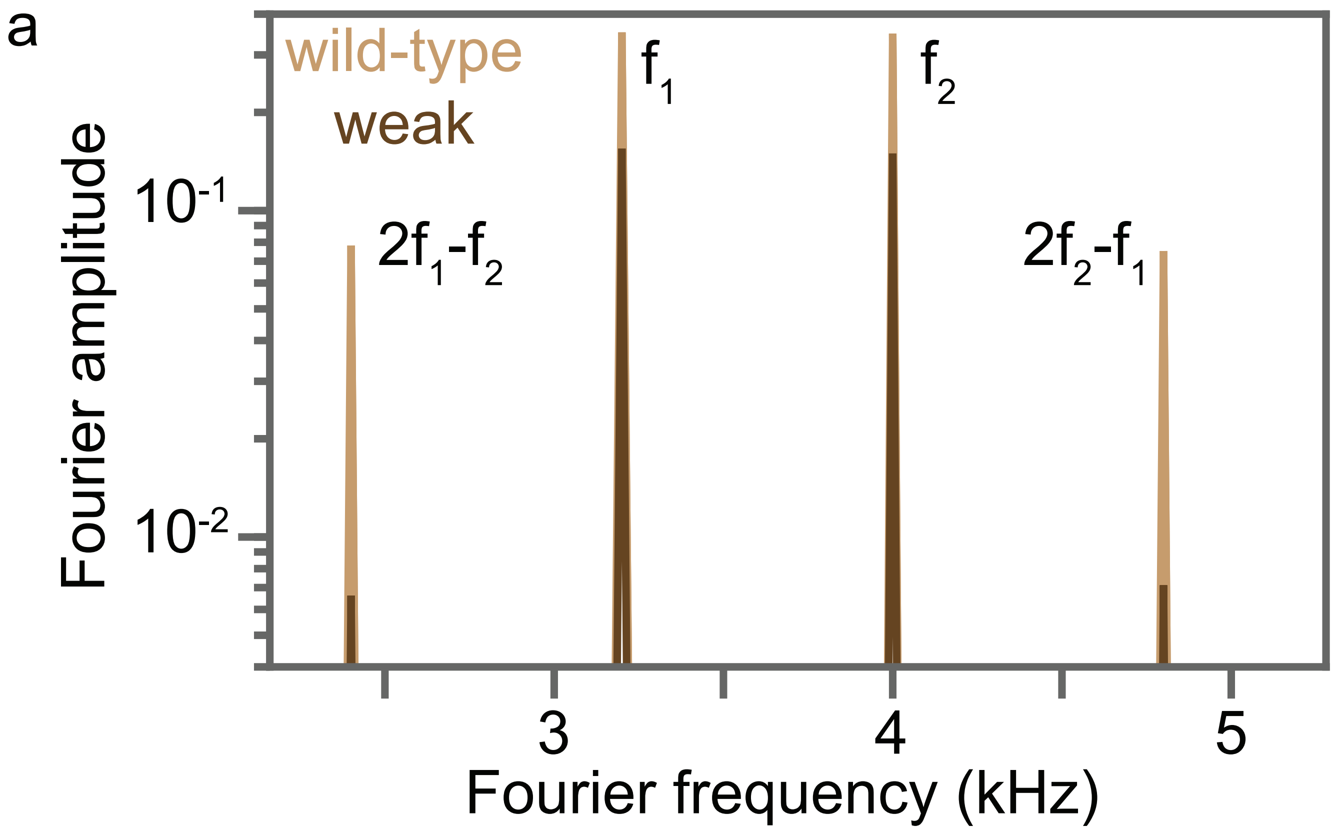

Figure S2. Distortion products are smaller in the weak gating-spring mutant. (a) A stimulus force of the form $F_1 \sin(2\pi f_1 t) + F_2 \sin(2\pi f_2 t)$ is applied in the model OHBs, in which $F_1 = F_2 = 490$ pN are the amplitudes of the total force applied to the OHB, $f_2 = 4$ kHz, and $f_1 = f_2/1.25 = 3.2$ kHz. Fourier-transform amplitudes of the receptor current are shown versus the Fourier frequency for the wild-type OHB (light brown) and the weak gating-spring mutant (dark brown). The receptor-current amplitudes at the primary frequencies (f_1 = 3.2 kHz, 0.35 for wild-type and 0.15 for weak gating-spring mutant; $f_2 = 4$ kHz, 0.35 for wild-type and 0.15 for weak gating-spring mutant) and at the cubic distortion product frequencies ($2f_1 - f_2 = 2.4$ kHz, 0.078 for wild-type and 0.0066 for gating-spring mutant; $2f_2 - f_1 = 4.8$ kHz, 0.075 for wild-type and 0.0071 for gating-spring mutant) are shown. The ratios of the distortion product amplitudes and the f_2 amplitude are larger for the wild-type OHB ($2f_1 - f_2$ ratio is $0.078/0.35 = 0.22$; $2f_2 - f_1$ ratio is $0.075/0.35 = 0.22$) than for the weak gating-spring mutant OHB ($2f_1 - f_2$ ratio is $0.0066/0.15 = 0.044$; $2f_2 - f_1$ ratio is $0.0071/0.15 = 0.048$).
